# Ethnomedicinal, Phytochemical and Pharmacological Investigations of *Perilla frutescens* (L.) Britt.

**DOI:** 10.3390/molecules24010102

**Published:** 2018-12-28

**Authors:** Hiwa M. Ahmed

**Affiliations:** Sulaimani Polytechnic University, Slemani 46001, Kurdistan Regional Government, Iraq; hiwa2009@yahoo.com or hiwa.ahmed@spu.edu.iq

**Keywords:** bioactivity, essential oils, polyphenols, preclinical, rosmarinic acid, terpeniods

## Abstract

*Perilla frutescens* (L.) Britt. (PF) is an annual herbal medicinal, aromatic, functional food, and ornamental plant that belongs to the mint family, Lamiaceae. The origin of perilla traces back to East Asian countries (China, Japan, Korea, Taiwan, Vietnam, and India), where it has been used as a valuable source of culinary and traditional medicinal uses. The leaves, seeds, and stems of *P. frutescens* are used for various therapeutic applications in folk medicine. In the absence of a comprehensive review regarding all aspects of perilla, this review aims to present an overview pertaining to the botanical drug, ethnobotany, phytochemistry, and biological activity. It was found that the taxonomic classification of perilla species is quite confused, and the number of species is vague. Perilla has traditionally been prescribed to treat depression-related disease, anxiety, asthma, chest stuffiness, vomiting, coughs, colds, flus, phlegm, tumors, allergies, intoxication, fever, headache, stuffy nose, constipation, abdominal pain, and indigestion, and acts as an analgesic, anti-abortive agent, and a sedative. Until now, 271 natural molecules have been identified in perilla organs including phenolic acids, flavonoids, essential oils, triterpenes, carotenoids, phytosterols, fatty acids, tocopherols, and policosanols. In addition to solvent extracts, these individual compounds (rosmarinic acid, perillaldehyde, luteolin, apigenin, tormentic acid, and isoegomaketone) have attracted researchers’ interest for its pharmacological properties. Perilla showed various biological activities such as antioxidant, antimicrobial, anti-allergic, antidepressant, anti-inflammatory, anticancer, and neuroprotection effects. Although the results are promising in preclinical studies (in vitro and in vivo), clinical studies are insufficient; therefore, further study needs to be done to validate its therapeutic effects and to ensure its safety and efficacy.

## 1. Introduction

*Perilla frutescens* (L.) Britt. is an annual herbal plant that belongs to the mint family, Lamiaceae [1,2]. It is commonly called perilla [3] or by other names (beefsteak plant, purple mint, perilla mint, Chines basil, Korean perilla, zisu in China, shiso in Japan, and tia to in Vietnam) [4]. It is widely cultivated throughout Asian countries such as China, Japan, South Korea, Vietnam, and India; however, China is probably considered to be a primary gene center for this species [1,2]. Perilla is historically an important herb that has been recorded in Chinese medical classics since around 500 A.D., especially in records named “Ming Yi Bie Lu” (Renown Physicians’ Extra Records), and others where the herb is registered as a drug named “su” which means comforting the body and promoting the blood circulation. From the ancient times in the Song dynasty (960–1279 A.D.), it can be seen that the stem, leaf, and seed of the herb was equally commonly used. The drug items of the herb in traditional Chinese medicine are dried “Perilla leaf”, “Perilla stalk” and “Perilla seed” corresponding to Folium Perillae, Caulis Perillae, and Fructus Perillae in the Chinese Pharmacopoeia (1990), while in the Japanese Pharmacopoeia (1991), Herba Perillae is listed as a drug derived from the leaves and twigs of perilla [5]. It has commonly been used as a traditional medicine and a functional food throughout Asian communities [6]. In the Chinese Pharmacopeia 2010, the dried parts of *P. frutescens*, such as stems Perillae Caulis (PCa), leaves Perillae Folium (PFo), and ripe fruits Perillae Fructus (PFr) are recorded for various therapeutic applications [4,7]. It has been used as a natural herbal medicine to recover from different symptoms, such as depression-related disease, asthma, anxiety, tumors, coughs, allergies, intoxication, cold, fever, chills, headache, stuffy nose, and some intestinal disorders [1,4,8,9,10]. It is used as an ornamental plant in gardens due to its wide morphological variability and attractive appearance [2]. It is also used as a kitchen herb in salads, sushi, soups, and as a spice, garnish, or food colorant as well. The seed oil is traditionally used to flavor foods [11]. Perilla also gains market importance in cosmetics, being processed in skin creams, soaps, and dermatological medicinal preparations, because of its biological activities [12]. The historical popularity and ethnopharmacological uses of this plant had attracted the interest of scientists to examine their pharmacological properties and resulted in a growing popularity among European countries as well. Interestingly, there are not many comprehensive scientific reviews to cover all aspects of information about perilla. Therefore, the purpose of this study is to provide an up-to-date summary in relation to the botanical, biological, phytochemical properties, and traditional uses in parallel with new perspectives of *P. frutescens*, and to provide an overview for future research on this plant.

## 2. Methodology

This review article was carried out to collect data from multiple databases, including PubMed, Web of Science, Wiley, Science Direct, Elsevier, American Chemical Society publications, SciFinder, and Google Scholar up to and including the year of 2018. The quality of the reviewed studies is well known, and only peer-reviewed articles were included, and considering only English literature that were available in the database; none of the studies in languages other than English were included for this review. No masters theses or Ph.D. dissertations, as well as unpublished articles, were included in this review. The pharmacological activities of perilla spices were reported only based on the extracted/isolated compounds directly found from perilla species, not commercially available unless otherwise stated, with appropriate controls in experiments. In silico study was not found in the literature and therefore excluded.

## 3. Botanical Characteristics and Aspects of Cultivation

The plant is a freely branching annual herb that grows up to 1.5 m high in some varieties. Stems are four-sided and covered with short hairs. Leaves are ovate, opposite, green to purple with toothed, crisped, laciniate, palmate, or serrate margins. They are glossy and downy-haired. The herb has a distinctive musky, mint-like odor. Flowers are small, bell-shaped, and a white or purple color with a distinctive ring of fine hairs along the bottom in terminal spikes or emerging from leaf axils, and four stamens are present in most species in that family with a gray-brown fruit [2,13]. The seeds are small and globular, and their 1000-seeds weight is about 4 g [3]. Its spreading is assured either by dropping close to the parent plant or they may be transported by wind or water. Perilla is said to resemble basil and coleus and may be confused with other members of the mint family.

According to Brenner [14] and Asif [3], the best diagnostic characteristics of perilla are the net-patterned testa and the distinctive smell of the crushed foliage. Perilla is reproduced via seeds. The cultivated varieties are generally grown using direct sowing or raised in nursery beds for transplanting in mid-spring. The optimal germination temperature is 20 °C, while it can be grown at slightly lower temperatures. The germination rate is fast and seed viability is lost relatively quickly over time; hence, in practice, fresh seeds are suggested to be used [2,13]. Perilla is a selfing [15], and a short-day plant in order to flower [16]. Harvesting is usually started at the end of September and the beginning of October [3], and this varies according to the intended use of the crop and climate condition in the area.

## 4. Classification and Taxonomy of Perilla

The taxonomic classification and nomenclature of perilla seems to be controversial and different systems have been published [14]. Based on the decoration pattern and size of pollen grains, in China, cultivated taxa of perilla could be divided into five varieties: var. *frutescens*, var. *arguta*, var. *crispa*, var. *auriculato*-*dentata*, and var. *acuta*. From those varieties, the leaves of var. *frutescens* and var. *acuta* are usually used as fresh vegetables and to process pickles, var. *crispa* is used for its medicinal properties, and seeds of var. *arguta* are used for oil extraction because of its high seed yield [17]. It is believed that, based on morphological characters and uses, the genus of *Perilla* L. consists of only one species and two diverse cultivars, such as *P. frutescens* (L.) Britton var. *frutescens* as an oil seed crop and *P. frutescens* (L.) Britton var. *crispa* (Thunb.) W. Deane as a spicy vegetable and medicine, which are cross-fertile (in both varieties, phenotypes with green and purple shoots can be found) [2]. According to *The Plant List* (www.theplantlist.org), three varieties—var. *frutescens*, var. *crispa* (Thunb.) H.Deane, and var. *hirtella* (Nakai) Makino—are the accepted species. The anthocyanin-rich purple colored types are frequently used as food colorants in Japan and China [12].

## 5. Ethnobotanical Uses

Perilla has been one of the most popular species in the Eastern Asian communities, that has been used as an ingredient, for flavor and as a spice in cooking, garnish, soups, vegetable salad, sushi, as a food colorant, and to wrap and eat cooked food in Japan, India, and Korea [4,18,19]. In Korea, perilla seed oil is used for cooking and different industrial uses [18]. Moreover, seeds as spice also used to prepare sauce in India [19]. As an antidote, perilla leaf has been used in fish and crab dishes in China and Japan for a long time [9]. In India, the whole plant has been used to treat stomach disorders and for flavoring curries, and in combination with *Artemisia scoparia* is used as a refrigerant [20]. The seed is used for meat preservation and flavoring foods [21]. Its seed oil is used to massage twice a day for arthritis [22], used for earache in Nepal [23], as well as a condiment and in food preparation of rice cakes [24,25]. Leaves are used for cooking as vegetables [24]. In Vietnam, the leaves are used as a spice [26], while in China and Thailand, it is used in temperature regulation in the form of hot infusion (tea) [27]. Leaf juice is used to expel intestinal worms and cut wounds in Dekhatbhuli, Nepal [23]. Perilla root paste mixed with goat urine is used as a poultice for rheumatoid arthritis twice daily for one week [28].

In Japan, it has historically been used for making drying oil for waterproofing umbrellas or as lamp oil. Perilla is used as a culinary herb to color and flavor pickles, as well as a garnish for raw fish [29]. In Indochina, especially in towns, perilla leaves are used as a vegetable, while in mountainous areas and countryside, the leaves are not exploited but mericarps are eaten. The seeds of perilla (mericarps) are also used like sesame seeds. Perilla mericarps are roasted and mixed into steamed sticky rice often with cane sugar [29].

In human traditional herbal medicine in China and India, the stem of the plant is historically used as an analgesic and anti-abortion agent. The leaves are believed to be useful against asthma, colds, flu, chest stuffiness, vomiting, cough, constipation, and abdominal pain, as well as promoting stomach function [19,30]. According to the Chinese traditions, *P. frutescens* could be used to cure a number of conditions such as cold, fever, chills, headache, and stuffy nose [4]. In addition, the plant has traditionally been prescribed to treat depression-related disease, asthma, anxiety, tumors, coughs, allergies, intoxication, and some intestinal disorders [1,8,9,10].

The juice of the fresh leaves is utilized for curing injuries and the seed oil for massaging infants [19]. Other indications for using the leaves include dissipating colds, promoting the circulation of Qi [4], toning the stomach, and detoxification [4,9]. According to some further references, the stem of PF is used for promoting the circulation of Qi [4], pain relief, and preventing miscarriage [19,30]. The seed of perilla seems to exhibit useful properties too. Yu et al. [4] describe its activity in descending Qi and resolving phlegm, relieving cough and asthma, and loosening the bowel to relieve constipation. Perilla leaf is mentioned as an ingredient in many Chinese herbal preparations, such as “Ban Xia Hou Po” decoctions used against discomfort in the throat, and as an essential herbal remedy for psychological disorders such as generalized anxiety.

## 6. Phytochemical Compounds in Perilla

**Figure 1:**
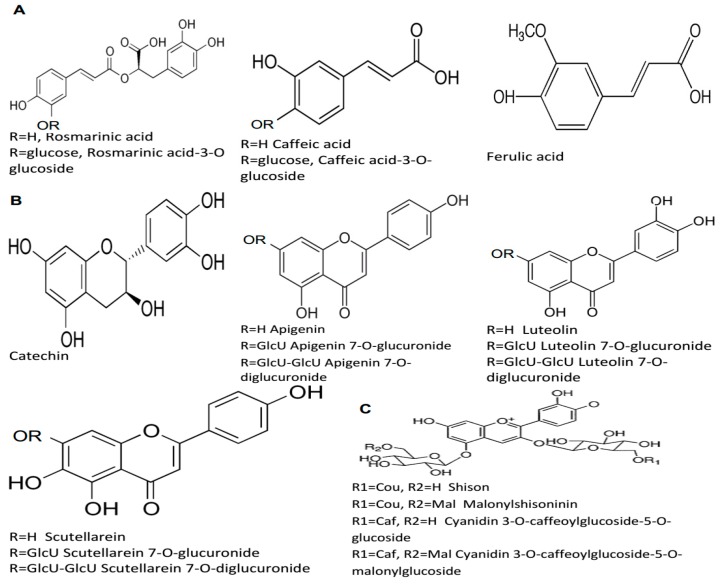
The chemical structures of chief hydrophilic compounds identified in *P. frutescens*: (**A**) phenolic acids; rosmarinic acid, rosmarinic acid-3-*O*-glucoside, caffeic acid, caffeic acid-3-*O*-glucoside, ferulic acid (**B**) flavonoids; catechin, apigenin, apigenin 7-*O*-glucuronide, apigenin 7-*O*-diglucuronide, luteolin, luteolin 7-*O*-glucuronide, luteolin 7-*O*-diglucuronide, scutellarein, scutellarein 7-*O*-glucuronide, scutellarein 7-*O*-diglucuronide, and (**C**) anthocyanins; shisonin, malonylshisonin, cyanidin 3-*O*-caffeoylglucoside-5-*O*-glucoside, cyanidin 3-*O*-caffeoylglucoside-5-*O*-malonylglucoside.

**Figure 2:**
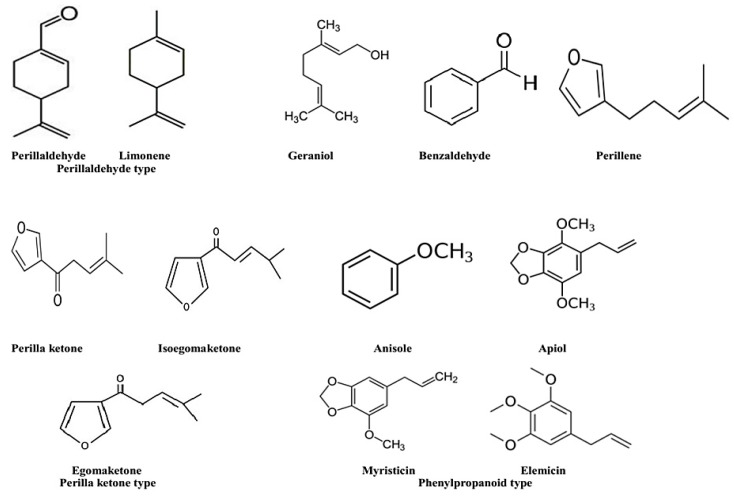
The chemical structures of some volatile compounds identified in *P. frutescens*; perillaldehyde, limonene, geraniol, perillene, benzaldehyde, perilla ketone, isoegomaketone, anisole, apiol, egomaketone, myristicin, elemicin.

**Figure 3:**
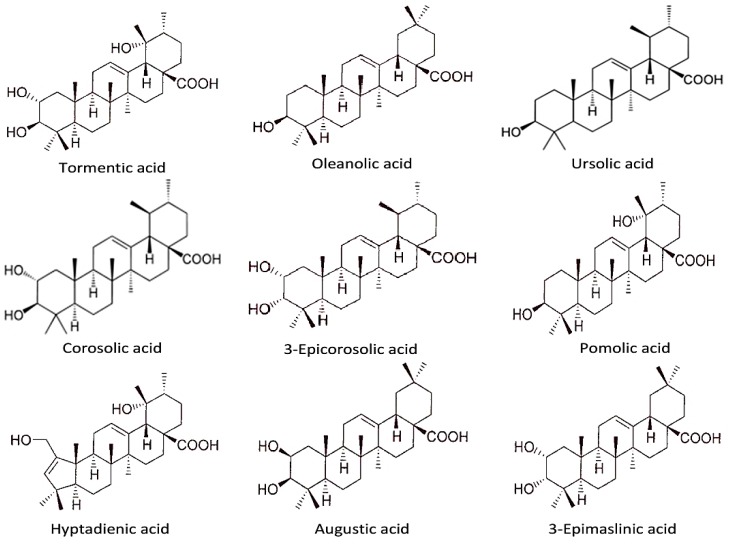
The chemical structures of triterpene acids identified in *P. frutescens*; tormentic acid, oleanolic acid, ursolic acid, corosolic acid, 3-Epicorosolic acid, pomolic acid, hyptadienic acid, augustic acid, 3-Epimaslinic acid.

**Figure 4:**
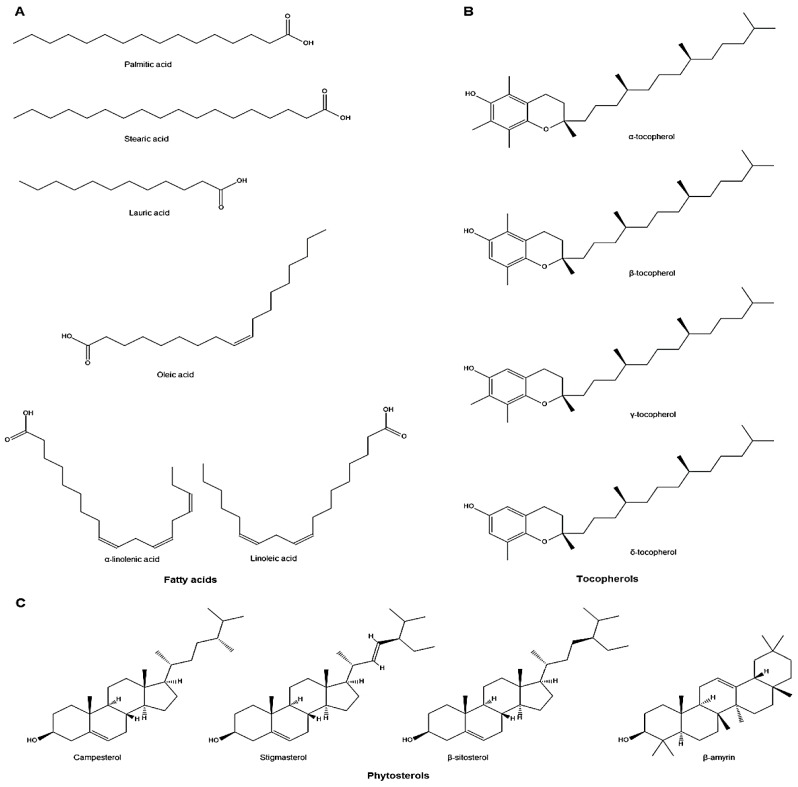
The chemical structures of major hydrophobic compounds identified in *P. frutescens*: (**A**) fatty acids; palmitic acid, stearic acid, lauric acid, oleic acid, linoleic acid, linolenic acid (**B**) tocopherols; α-tocopherol, β-tocopherol, γ-tocopherol, δ-tocopherol and (**C**) phytosterols; campesterol, stigmasterol, β-sitosterol, β-amyrin.

There are currently 271 various phytochemical compounds that have been isolated and reported in perilla seeds, stems, and leaves. Based on the chemical properties, these active compounds in perilla could be classified either as hydrophilic (phenolic acids, flavonoids, anthocyanins) or hydrophobic (lipophilic) ones (volatile compounds, triterpenes, phytosterols, fatty acids, tocopherols, and policosanols). The identified phytochemicals are listed in Table 1, Table 2 and Table 3, and some of their structures are displayed in Figure 1, Figure 2, Figure 3 and Figure 4.

### 6.1. Phenolic Compounds, Flavonoids, and Anthocyanins

Phenolic compounds frequently occur in the perilla plant. They have a wide structural variability with a broad range of pharmacological activities (Table 1, Figure 1). Peng et al. [31] reported for the first time that catechin, ferulic acid, apigenin, luteolin, rosmarinic acid, and caffeic acid are determined by capillary electrophoresis in leaves and seeds of perilla. Similarly, Gu et al. [32] identified rosmarinic acid, luteolin, apigenin, and chrysoeriol by means of ultraviolet–visible spectroscopy, nuclear magnetic resonance spectroscopy (NMR) and electrospray ionisation mass spectrometry (ESI-MS) from the fruit of *P. frutescens* var. *acuta*. Meng et al. [11] identified various polyphenols from different varieties of perilla (var. *crispa* and var. *frutescens*) Britt., which included cinnamic acid derivatives (coumaroyl tartaric acid, caffeic acid, rosmarinic acid), flavonoids (apigenin 7-*O*-caffeoylglucoside, scutellarein 7-*O*-diglucuronide, luteolin 7-*O*-diglucuronide, apigenin 7-*O*-diglucuronide, luteolin 7-*O*-glucuronide, scutellarein 7-*O*-glucuronide), and anthocyanins (mainly cis-shisonin, shisonin, malonylshisonin and cyanidin 3-*O*-(E)-caffeoylglucoside-5-*O*-malonylglucoside). Zhou et al. [1] identified eleven phenolic compounds from cold-pressed *P. frutescens* var. arguta seed using column chromatography. These compounds were partly identical with the previously mentioned results: 30-dehydroxyl-rosmarinic acid-3-*O*-glucoside, rosmarinic acid-3-oglucoside, rosmarinic acid, rosmarinic acid methyl ester, luteolin, luteolin-5-*O*-glucoside, apigenin, caffeic acid, caffeic acid-3-*O*-glucoside, vanillic acid, and cimidahurinine using ion-trap time-of-flight mass spectrometry (IT-TOF MS) and nuclear magnetic resonance (NMR) analyses. Rosmarinic acid has been reported to be one of the chief phenolic compounds in perilla leaves [7] and recent study [33], showed more accumulation of phenolic components at the full flowering stage. It has been shown that the red color was given by the presence of a major anthocyanin, malonylshisonin, 3-*O*-(6-*O*-(E)-p-coumaryl-β-d-glucopyranosyl)-5-*O*-(6-*O*-malonyl-β-d-glucopyranosyl)-cyanidin [12], and other related anthocyanin compounds that accumulate in the epidermal cells of leaves and stems of the red-leaf chemotype [34]. The green-leaf chemotypes show only trace amounts of anthocyanin type compounds among all polyphenol compounds encountered [12].

### 6.2. Volatile Compounds

Essential oils (EOs) are a type of secondary metabolites that can be extracted from different aromatic plant organs such as flowers, buds, stems, bark, leaves, fruits, etc. [36]. Their most frequent components are terpenoids, and aromatic and aliphatic compounds. A number of varieties are distinguished by the various chemical compositions of the essential oils extracted from their plant organs as a primary component of the oil known as chemotypes. According to Ito, Zhang, and Ghimire et al. [37,38,39], based on the main components of the EOs, different chemotypes have been described in perilla such as perillaketone (PK) (isoegomaketone), perillaldehyde (PA), elsholtziaketone (EK), citral (C), phenylpropanoids (PP) (myristicin, dillapiole, elemicin), perillene (PL), beta-caryophyllene, myristicine (MT), limonene, and piperitenone (PT). EOs are known to possess various bioactivities including antibacterial, antiviral, antifungal, anti-inflammatory, antimutagenic, anticarcinogenic, antidiabetic, antiprotozoal, and antioxidant [36].

The composition of EO of different organs of the perilla plant has been frequently analyzed and, until present, 193 different compounds have been identified (Table 2, Figure 2). The method of extraction may have a significant effect on the composition of the extracts too. Huang et al. [40] compared hydrodistillation, supercritical fluid extraction (SFE-CO2), and headspace solid phase microextraction (HS-SPME), followed by gas chromatography-mass spectrometry (GC–MS) analysis of volatile compounds and they found 64 compounds, mainly perillaldehyde and perilla ketone. Tian et al. [41] identified 119 compounds from the essential oil of perilla from eleven different areas, of which the predominant compounds were 2-acetylfuran (max. 82.17%), perillaldehyde (max. 53.41%), caryophyllene (max. 38.34%), laurolene (max. 40.6%), 2-hexanoylfuran (max. 33.03%), 2-butylamine (max. 22.22%), α-asarone (max. 11.85%), farnesene (max. 9.25%), α-caryophyllene (max. 9.16%), and (Z,E)-farnesene (max. 7.14%). Sixty-five aromatic compounds were identified from ten perilla accessions with the predominance of perillaldehyde, perilla ketone, β-dehydro-elsholtzia ketone, limonene, shisofuran, farnesene (Z, E, α), β-caryophyllene, and trans-shisool [42].

### 6.3. Other Terpenoids

Carotenoids are organic pigments, which belong to the category of tetraterpenoids and are widely distributed in nature, accumulating in chloroplasts. Perilla showed higher carotenoid content, even compared to β-carotene-rich (carrots, spinach) or lutein-rich (spinach, broccoli, lettuce) crops, where the content of carotenoids in perilla is up to five-fold higher [48]. Moreover, trace triterpenes (Table 3, Figure 3), including tormentic acid, oleanolic acid, and ursolic acid, were determined in perilla using high-performance liquid chromatography (HPLC) analysis [50]. There are also some phytosterol (Table 3, Figure 4) compounds (ampesterol, stigmasterol, β-sitosterol, β-amyrin, oxalic acid, triacylglycerols) that have been found in perilla seeds. The content of β-sitosterol was demonstrated to definitely correlate to the content of linolenic acid [51].

### 6.4. Fatty Acids and Other Lipid Type Compounds

Perilla oil constitutes roughly 40% of the seed weight, and seeds of perilla are a good source of fatty acid composition such as palmitic acid (C17:0), stearic acid (C18:0), oleic acid (C18:1), linoleic acid (C18:2), and linolenic acid (C18:3). In addition, the content of unsaturated fatty acids in perilla seed oils is typically over 90%, contains considerably high levels of α-linolenic acid (ω-3 fatty acid) (α–LNA) ranges from 52.58% to 61.98%. Furthermore, the ω-6 (linoleic acid) around 14% and ω-9 (oleic acid) is also present in perilla oil. These polyunsaturated fatty acids are expected to possess various health benefits for humans such as reducing the cholesterol and triglyceride levels in serum, lowering the risk of colon cancer, and preventing the excessive growth of visceral adipose tissue [3,52,53]. There are also four types of tocopherols (α-, β-, γ-, and δ-tocopherol) that have been found in PF seeds. The content of γ-tocopherol was demonstrated to definitely correlate to the content of linolenic acid [51] (Table 3, Figure 4).

### 6.5. Policosanols

Policosanols are very long chain aliphatic alcohols derived from the wax constituent of plants. Contents and compositions of the waxy materials and policosanols (Table 3) were identified and quantified using thin layer chromatography (TLC), HPLC, and GC. Waxy materials, moisture, and crude lipids from perilla seeds comprised about 72 mg/100 g, 5.6–8.2%, and 51.2–48.4%, respectively, and major components of waxy materials were policosanols (25.5–34.8%), wax esters, steryl esters, and aldehydes (53.0–49.8%), hydrocarbons (18.8–10.5%), acids (1.7–2.1%), and triacylglycerols (1.0–2.9%), analyzed using HPLC. Policosanols extracted in the waxy materials of the PF seeds were also determined based on gas chromatography to be 67–68% octacosanol, 16–17% hexacosanol, and 6–9% triacontanol of the total policosanols composition [56]. The seed of perilla was found to be rich in policosanols with 427.83 mg/kg of oil [59].

### 6.6. Nutrients

The good nutritional value, such as ash content 2.2%, crude fiber 23.28%, crude protein 5.12%, carbohydrates 18.53%, and minerals like calcium 0.238, magnesium 0.325, potassium 0.5004, and phosphorus 0.2124 (mg/g), and the fatty acid composition of perilla seed, was also reported [30]. The quality of protein from perilla seed was investigated and an excellent amino acid profile of perilla seed protein was found [60].

## 7. Pharmacological Properties of Perilla

As mentioned earlier, the biological activity of perilla is due to the presence of various biochemical compounds that are responsible for producing health benefits for humans. Because of this, many researchers have focused on the isolation and identification of these active ingredients as well as their biological evaluations.

### 7.1. Antioxidant Activity

Epidemiological, clinical, and nutritional studies show that consumption of so-called functional foods and nutraceuticals may be associated with a lowered risk of cancers, cardiovascular diseases, and metabolic disorders [61]. These benefits are often attributed to the high antioxidant capacity of the drug, and especially to the content of phenolic acids, flavonoids, and carotenoids. It has been reported that extracts from perilla seeds and leaves exhibit concentration-dependent antioxidant activity, based on the 2,2-diphenyl-1-picryl-hydrazyl-hydrate (DPPH) radical assay, and 2,2′-azino-bis(3-ethylbenzothiazoline-6 sulphonic acid) (ABTS) radical cation assay [1]. Isolated rosmarinic acid (RA) and luteolin from the fruit of *P. frutescens* var. *acuta* showed significant DPPH scavenging capacity with half-maximal inhibitory concentration (IC_50_) values of 8.61 and 7.50 µM, respectively [32]. Similarly, among five phenolic compounds, RA and rosmarinic acid-3-*O*-glucoside were the dominant phenolic antioxidants with strong activity from cold-pressed perilla var. *arguta* seed flour studied by Zhou et al. [1]. RA isolated by these authors from perilla leaf exhibited DPPH radical scavenging activity of 88.3 ± 0.7% at a concentration of 10 μg/mL with a drug concentration eliciting 50% of the maximum stimulation (SC50) value of 5.5 ± 0.2 μg/mL. Tian et al. [41] proved that the antioxidant activity of perilla essential oil may depend on the location of cultivation. Extracts of drugs harvested from different regions exhibited varying degrees of scavenging ability at 10 mg/mL concentrations with an inhibition percentage of 94.80 ± 0.03%. The 80% methanol extract of perilla seeds exhibited a strong antioxidant property [62]. In vivo, the protective activity of RA from *P. frutescens* leaf (PFL) was demonstrated on Lipopolysaccharide (LPS)-induced liver injury of d-GalN-sensitized mice owing to the scavenging or reducing activities of superoxide or peroxynitrite rather than to inhibition of tumor necrosis factor (TNF)-α production [63].

The roles of the flavonoid luteolin from the perilla seeds seems to provide significant antioxidant activity for drugs and extracts. This compound significantly reversed hydrogen peroxide-induced cytotoxicity in primary cultured cortical neurons. Whereas, luteolin markedly attenuated the reactive oxygen species (ROS) production, and prevented the decrease in activities of mitochondria, catalase, and glutathione in ROS-insulted primary neurons, for preventing neurodegenerative diseases [64]. In another study, luteolin inhibited the peroxidation of linoleic acid catalyzed using soybean lipoxygenase-1 with an IC_50_ of 5.0 M (1.43 μg/mL) noncompetitively [65].

The monoterpene perillaldehyde was shown to be a potent thioredoxin inducer as it activates the Nrf2-Keap1 system [66]. It seems that the antioxidant activity of perilla may vary among different accessions. As part of an in vitro study in a human subjects, purple perilla leaves showed a higher antioxidant activity, and prevented the oxidation of low-density lipoprotein (LDL) than the green leaves [67]. Another study revealed that 2′,3′-dihydroxy-4′,6′-dimethoxychalcone (DDC) found in green perilla leaves enhanced cellular resistance to oxidative damage through activation of the Nrf2-antioxidant response element (ARE) pathway [68].

### 7.2. Antibacterial and Antifungal Activity

Recently, the demand for natural compounds from plant extracts as effective antibacterial agents against a wide range of bacteria is definitely growing to control human infection and for the preservation of food [69]. Perilla seed extract rich in polyphenols was examined for its antibacterial activity against oral cariogenic *Streptococci* and periodontopathic *Porphyromonas gingivalis*. The ethyl acetate extracts exhibit strong antibacterial activity against oral *Streptococci* and various strains of *P. gingivalis*. On the other hand, the ethanolic extract of defatted perilla seed weakly inhibited the growth of oral pathogenic bacterial strains. Among the polyphenols, luteolin showed marked antibacterial activity against the oral bacteria tested [70]. Additionally, the antibacterial activity of the essential oil from perilla leaves on Gram-positive and Gram-negative bacteria was studied, and the results showed the effectiveness of this essential oil to inhibit the growth of the tested bacteria. The minimum inhibitory concentration (MIC) on *Staphylococcus aureus* and *Escherichia coli* were 500 μg/mL and 1250 μg/mL. respectively [71]. The most abundant terpene-type compound, perillaldehyde, moderately inhibits a broad range of both bacteria in the range of 125–1000 pg/mL. This compound was also particularly active against filament fungi, with MIC values for *M. mucedo* and *P. chrysogenum* already at a 62.5 pg/mL concentration [72]. Kim and Choi [69,73] determined the antibacterial activity of the leaf ethanol extracts of PF var. *acuta* against *S. aureus* and *Pseudomonas aeruginosa* and detected that the population of *P. aeruginosa* decreased from 6.660 to 4.060 log CFU/mL, and that of *S. aureus* from 7.535 to 4.865 log CFU/mL, as well as to 2.600 log CFU/mL via extraction with ethyl acetate.

The fungicidal effects of perilla EO were described against *Trichophyton mentagrophytes* [74], and they dose-dependently decreased the production of α-toxin, enterotoxins A and B, and toxic shock syndrome toxin 1 (TSST-1) in both methicillin-sensitive *S. aureus* and methicillin-resistant *S. aureus* [75]. The antifungal activity of perilla EO distilled from aerial parts of the plant was also tested against phytopathogenic fungi and its activity was demonstrated in the cases of *Aspergillus flavus*, *Aspergillus oryzae*, *Aspergillus niger*, *Rhizopus oryzae*, and *Alternaria alternate* [41].

### 7.3. Anti-Allergic Effect

Studies show that water extracts of PF may inhibit allergic reactions in vivo and in vitro. PF extracts (0.05 to 1 g/kg) greatly inhibited systemic allergic reactions activated by anti-DNP IgE in rats in a dose-dependent manner [76]. Similarly, the water extract of PFL has been shown to have a positive result against atopic dermatitis in an animal model. The anti-allergic effects of PFL on 2,4-dinitrofluorobenzene (DNFB)-induced atopic dermatitis in C57BL/6 mice was evaluated by Heo et al. [77] and the results revealed that an aqueous extract (100 μg/mL) of PFL could significantly inhibit DNFB-induced atopic inflammation by alleviating the expression of MMP-9 and IL-31, as well as augmenting T-bet activity. In another experiment, water extract from PFL significantly suppressed the PCA-reaction, using mice ear-passive cutaneous anaphylaxis (PCA)-reaction, and the authors concluded the role of rosmarinic acid [9]. Application of an ethanol extract from PF, rather than the aqueous extract, suppressed the allergen-specific Th2 responses. Furthermore, airway inflammation and hyperreactivity in an ovoalbumin-sensitized murine model of asthma were alleviated. Based on this, Chen et al. [78] suggested perilla as a potential phytotherapeutic tool for immunomodulation.

Besides using crude extracts, individual compounds as a potential biologically active agent against allergies have also been studied. A novel glycoprotein fraction from the hot water extract of perilla was used and it was found that it moderately inhibited mast cell degranulation and the activities of hyaluronidase (IC_50_ = 0.42 mg/mL) in a dose-dependent manner [79]. Furthermore, daily oral supplementation with RA (1.5 mg/mouse, orally) from perilla significantly prevented the increase in the numbers of eosinophils in bronchoalveolar lavage fluids and in those around murine airways. Likewise, the expression of IL-4 and IL-5, and eotaxin in the lungs of sensitized mice, together with allergen-specific IgG1, were also inhibited. Due to these findings, the authors revealed RA as an effective intervention against allergic asthma [80]. In other study, perilla extracts enriched with RA could inhibit seasonal allergic rhinoconjunctivitis in humans at least partly via inhibition of polymorphonuclear leukocytes (PMNL) infiltration into the nostrils [81]. The use of a diet supplemented with perilla oil might be effective on asthmatic allergy via decreasing serum lipids and ovalbumin-specific IgG1 and IgA levels in mice [82].

### 7.4. Anti-Depressant Activity

Numerous studies focusing on the extracts and/or purified compounds of *P. frutescens* displayed antidepressant-like effects. Phenolic-type constituents of perilla leaf, such as apigenin, at intraperitoneal doses of 12.5 and 25 mg/kg [83], RA (2 mg/kg, i.p.) and caffeic acid (4 mg/kg, i.p.) each led to a considerable reduction of the duration of immobility in the forced swimming test. These compounds are also supposed to inhibit the emotional abnormality produced by stress [84,85], which is possibly mediated by the dopaminergic mechanisms in the mouse brain [83].

Essential oils and perillaldehyde from perilla leaves were also found to show an anti-depressant property in mice with CUMS-induced depression [86,87]. In another study, daily consumption of perillaldehyde (20 mg/kg, oral) demonstrated significant antidepressant-like effects in mice with LPS-induced depression and the authors concluded a potential benefit in inflammation-related depression [88]. Inhaling the same compound (perillaldehyde 0.0965 and 0.965 mg/mouse/day, 9 days) had antidepressant-like properties on a stress-induced, depression-like model in mice during the forced swim test (FST) through the olfactory nervous function [89].

The oil of PF seeds might have an anti-depressant activity too since a seed oil-rich diet during a forced swim test in adult male rats modulated the fatty acid profiles and brain-derived neurotrophic factor (BDNF) expression in the brain [90]. Moreover, perilla seed oil rich in *n*-3 fatty acids improved cognitive function in rats by generating new hippocampal neural membrane structures as well as by inducing specific protein expression [91].

### 7.5. Anti-Inflammatory Activity

Luteolin has been isolated from PFL ethanol extracts and was demonstrated to exert beneficial effects on neuro-inflammatory diseases in a dose-dependent manner (IC_50_ = 6.9 μM) through suppressing the expression of inducible nitric oxide synthase (iNOS) in BV-2 microglial cells [43].

The ethanol extract of PFL was identified to display significant anti-inflammatory activity in LPS-induced Raw 264.7 mouse macrophages through the inhibition of the expression of pro-inflammatory cytokines, inhibition of mitogen-activated protein kinase (MAPK) activation, and of nuclear factor-kappa (NF-κB) nuclear translocation in response to LPS [92]. The seed oil from PF showed a great protective effect against reflux esophagitis and this could be attributed to the antisecretory (anticholinergic, antihistaminic), antioxidant, and lipoxygenase inhibitory activities due to the presence of α-Linolenic acid (ALA) (18:3, *n*-3) [93]. Furthermore, RA isolated from PFL could inhibit the release of high mobility group box 1 protein (HMGB1) and down-regulated HMGB1-dependent inflammatory responses in human endothelial cells, HMGB1-mediated hyperpermeability, and leukocyte migration in mice, as well as reduced cecal ligation and puncture (CLP)-induced HMGB1 release and sepsis-related mortality. This could be a potential remediation for various vascular inflammatory diseases, such as sepsis and septic shock, via inhibition of the HMGB1 signaling pathway [94]. Lipophilic triterpene acids from ethanol extracts of red and green PFL were demonstrated to have remarkable anti-inflammatory activity on 12-*O*-tetradecanoylphorbol-13-acetate (TPA)-induced inflammation in mice (ID_50_: 0.09–0.3 mg per ear), and on the Epstein–Barr virus early antigen (EBV-EA) activation (91–93% inhibition at 1 × 10^3^ mol ratio/TPA), [54]. A recent study in mice showed that PF extract ameliorated inflammatory bowel disease (IBD) via protection of dextran sulfate sodium-induced murine colitis, with NF-κB and signal transduction and activator of transcription 3 (STAT3) as putative targets [95]. A perillaketone-type and alkaloid isolated from aerial parts of perilla showed the remarkable inhibitory effect on pro-inflammatory cytokines (TNF-α and/or IL-6) and inflammatory mediator (NO) in LPS-stimulated RAW264.7 cells, indicating that these compounds might be active components for inflammatory disorders [96].

### 7.6. Antitumor Effect

A number of in vivo and in vitro studies have reported the potential anticancer activity of PF. Tormentic acid, a lipophilic triterpene acid from ethanol extracts of red and green PFL, remarkably blocked carcinogenenesis in an in vivo, two-stage mouse skin model [54]. Similarly, in an in vivo carcinogenesis model, topical application of perilla-derived fraction (2.0 mg/mouse) led to a significant reduction of 7,12-dimethylbenz(a)anthracene (DMBA)-initiated and TPA-promoted tumorgenesis. This is probably based on two independent effects: inhibition of oxidative DNA injury and inhibition of adhesion molecule, chemokine, and eicosanoid synthesis [97].

In addition, Lin et al. [98] evaluated the inhibitory effects of PFL and they found that it effectively induces apoptosis-related genes and could inhibit cell proliferation in human hepatoma HepG2 cells. They also observed that the inhibitory effect of PFL was much higher than the same dose of commercially available RA and luteolin compounds.

In another study, the application of ethanol extract of PFL resulted in induced apoptosis through the combinations of death receptor-mediated, mitochondrial, and endoplasmic reticulum stress-induced pathways, and substantially suppressed the cell proliferation via p21-mediated G1 phase arrest in human leukemia HL-60 cells [99]. Isoegomaketone (IK), an essential oil component of PF, was found to be another potential agent possessing anti-cancer activity. IK induces apoptosis through caspase-dependent and caspase-independent pathways in human colon adenocarcinoma DLD-1 cells [100].

### 7.7. Miscellaneous Effects

In addition to the pharmacological activities described above, different extracts, seed oil, and some individual phenolic compounds of perilla have been found to exhibit other special physiological activities indicating further therapeutic utilizations.

An aqueous extract of PF showed potent anti-HIV-1 activity via inhibition giant cell formation in co-culture of Molt-4 cells with and without HIV-1 infection showing inhibitory activity against HIV-1 reverse transcriptase [101]. A very recent study indicates the importance of PFL extracts as a potential anti-aging agent for skin, as it showed effectiveness against UV-induced dermal matrix damage in vitro and in vivo [102]. The in vitro neuroprotection activity of unsaturated fatty acids of PF seed oil have been reported by Eckert et al. [103]. Perilla seed oil might be useful for other complaints too. Deng et al. [104] described in vitro and in vivo anti-asthmatic effects of perilla seed oil in the guinea pig and concluded that the oil may ameliorate lung function in asthma by regulating eicosanoid production and suppressing leukotriene (LT) generation. Zhao et al. [105] supposed a possible anti-ischemic activity of luteolin extracted from PFL, likely through a rebalancing of pro-oxidant/antioxidant status.

In vivo, the protective activity of RA from PFL was demonstrated on LPS-induced liver injury of d-GalN-sensitized mice. The treatments significantly reduced the elevation of plasma aspartate aminotransferase (AST) and alanine transaminase (ALT) levels, as well as anti-TNF and sphincter of Oddi dysfunction (SOD) treatment, compared with controls [63]. In one investigation, the hepatoprotective effects of sucrose-treated perilla leaves, other than untreated leaves, exhibited the best result in vitro and in vivo [10].

## 8. Toxicology

Although a well-established application of products from any herb including perilla require proofs not only for efficacy but also for safety. Suprisingly, there are only very few studies that have been reported about the toxicological aspects of materials originating from perilla. Inhaling smoke from roasting perilla seeds led to occupational asthma through an IgE-mediated mechanism [106]. Additionally, a single case of anaphylaxis caused by perilla seed was also reported [107].

## 9. Conclusions and Future Perspectives

*P. frutescens* L. varieties have a long traditional usage in many Asian countries and now across the world. The plant has been cultivated for multiple usages, traditionally for curing depression-related disease, asthma, anxiety, tumors, coughs, allergies, intoxication, cold, fever, chills, headache, stuffy nose, and some intestinal disorders, and acts as an antioxidant. Due to genetic variations, it has been exploited as an ornamental plant in gardens. Taxonomical aspects of perilla species must be recognized to avoid misleading identification of the plant species via a proper molecular study. The traditional and local uses of the plant are not well documented in the English literature since the plant originally belongs to the Asian countries, and might be the main reason why the ethnobotanical uses of perilla species are not widespread. In addition, it was also used as an edible aromatic vegetable plant to flavor foods. The leaves and seeds have high nutritional value since the leaf is rich in carotenoids and the seed is rich in fatty acid oils, and both have a potential use as functional dietary supplements in food industries. There are 271 active compounds have been reported including phenolic acids, flavonoids, triterpenes, volatile compounds, policosanols, carotenoids, fatty acids, tocopherols, and phytosterols. In addition to the crude solvent extracts, these phytochemicals (rosmarinic acid, perillaldehyde, luteolin, apigenin, tormentic acid, isoegomaketone) are the most studied natural compounds derived from perilla species.

Most of the pharmacological studies outlined in this review are in vitro and in vivo assays that can provide a basis for further studies. The traditional medicinal uses are well-correlated in terms of anti-allergy and anti-depressant activity in which they are claimed for. However, one of the active components of perilla essential oil is perilla ketone, which was found in varied quantities in several perilla species and other herbs. This compound has been shown by Kerr et al. [108] to possess pulmonary toxicity in some animals, e.g., horses, sheep, and cows, but there is no evidence for the toxicity of perilla ketone in humans [109]. Toxicological profiles of the active constitutes of perilla, especially aromatic compounds (essential oils), are significantly lacking and need to be addressed. Besides that, the clinical study is not much studied such that further research in this context is necessary to fully ascertain and understand the pharmacological activities and their mechanisms. Although its bioactivity in vitro and in vivo has been revealed to present potential health benefits, such as anti-microbial, antioxidant, anti-allergic, antidepressant, anti-inflammatory, anticancer, and neuroprotective effects, the clinical trials are insufficient to declare a well-established efficacy and safety, therefore human studies are recommended.

## Figures and Tables

**Table 1:** Phenolic acids, flavonoids, and anthocyanin compounds found in *Perilla frutescens* species.

Number	Compounds	Plant Organs	References
**Phenolic compounds**			
1	30-dehydroxyl-rosmarinic acid-3-*O*-Glucoside	Seeds	[1]
2	Caffeic acid	Leaves, Seeds	[1,11,31,35]
3	Caffeic acid-3-*O*-glucoside	Seeds	[1,8]
4	Coumaroyl tartaric acid	Leaves	[11]
5	Ferulic acid	Leaves, Seeds	[31]
6	Rosmarinic acid	Stems, Leaves, Seeds	[1,7,8,11,31,32,34,35]
7	Rosmarinic acid methyl ester	Seeds	[1,35]
8	Rosmarinic acid-3-*O*-glucoside	Seeds	[1,8]
9	Vanillic acid	Seeds	[1]
**Flavonoids**			
10	(+)-catechin	Leaves, Seeds	[31]
11	Apigenin	Leaves, Seeds	[1,8,31,32]
12	Apigenin 7-*O*-glucuronide	Leaves	[34]
13	Apigenin 7-*O*-caffeoylglucoside	Leaves	[11,34]
14	Apigenin 7-*O*-diglucuronide	Leaves	[11,34]
15	Catechin	Leaves, Seeds	[31]
16	Cimidahurinine	Seeds	[1]
17	Chrysoeriol	Seeds (fruits)	[32]
18	Luteolin	Leaves, Seeds	[1,8,31,32]
19	Luteolin 7-*O*-diglucuronide	Leaves	[11,34]
20	Luteolin 7-*O*-glucoside	Leaves	[34]
21	Luteolin 7-*O*-glucuronide	Leaves	[11]
22	Luteolin-5-*O*-glucoside	Seeds	[1]
23	Scutellarein	Leaves	[11,34]
24	Scutellarein 7-*O*-diglucuronide	Leaves	[11,34]
**Anthocyanins**			
25	Chrysontenin	Leaves	[35]
26	*cis*-Isomer of malonylshisonin	Leaves, Stems	[34]
27	*cis*-Shisonin	Leaves	[11]
28	Cyanidin 3-*O*-caffeoylglucoside-5-*O*-glucoside	Leaves, Stems	[34]
29	Cyanidin 3-*O*-[E]-caffeoylglucoside-5-*O*-malonylglucoside	Leaves	[11]
30	Cyanidin 3-*O*-caffeoylglucoside-5-*O*-malonylglucoside	Leaves, Stems	[34]
31	Cyanidin-3-*O*-(6-Ocaffeoyl)glucoside-5-*O*-glucoside	Leaves	[35]
32	Cyanidin-3-*O*-(6-Ocoumaroyl)glucoside	Leaves	[35]
33	Cyanidin-3-*O*-(6-Ocoumaroyl)glucoside-5-*O*-glucoside	Leaves	[35]
34	Cyanin	Leaves, Stems	[34,35]
35	Malonylshisonin	Leaves, Stems	[11,34]
36	Peonidin 3-*O*-malonylglucoside-5-*O*-*p*-coumarolglucoside	Leaves, Stems	[34]
37	Peonidin 3-*O*-malonylglucoside-5-*O*-*p*-coumarylglucoside	Leaves, Stems	[34]
38	Peonidin 3-*O*-malonylglucoside-5-*O*-*p*-coumarylglucoside	Leaves, Stems	[34]
39	Shisonin	Leaves, Stems	[11,34]

**Table 2:** Volatile oil compounds found in *Perilla frutescens* species.

Number	Compounds	Parts	References
40	(E,E)-α-Farnesene	Leaves	[40]
41	(Z)-3-Hexenyl acetate	Leaves	[40,43]
42	(Z,E)-α-Farnesene	Leaves	[40,41,44]
43	1-(3-Cyclohexen-1-yl)-2,2-dimethyl-1-propanone	Leaves	[40]
44	1,10-Decanediol	Leaves	[41]
45	1,2-Benzenedicarboxylic acid	Leaves	[7]
46	1,4,7,-Cycloundecatriene, 1,5,9,9-Tetramethyl-, Z,Z,Z-	Stems, Leaves, Seeds	[7]
47	1,6-Cyclodecadiene	Leaves	[7]
48	10-Undecyn-1-ol	Leaves	[41]
49	1-Cyclohexane-1-carboxaldehyde	Stems, Leaves, Seeds	[7]
50	1-Cyclohexene-1-methanol	Leaves	[41]
51	1-Octen-3-ol	Leaves	[40,41,43,44]
52	2,2-Dimethylpentane	Leaves	[41]
53	2,4,6-Triisopropylphenol	Leaves	[40]
54	2,4-Hexadienal	Leaves	[40,41]
55	2-Acetyl-5-methyl furan	Leaves	[43]
56	2-Acetylfuran	Leaves	[41]
57	2-Butylamine	Leaves	[41]
58	2-Cyclopentenone	Leaves	[41]
59	2-Ethyladamantane	Leaves	[40]
60	2-Hexanoylfuran	Stems, Leaves, Seeds	[7,41]
61	2-Hexenal	Leaves	[40,41]
62	2-Hexenal	Leaves	[43]
63	2-Hydroxypyridine	Leaves	[41]
64	2-Isopropylidene-3-methyl-hexa-3,5-dienal	Leaves	[40]
65	2-Methoxy-3-propenyl-phenol	Leaves	[7]
66	2-Methyl-2-cyclopentenone	Leaves	[41]
67	2-Methylcyclopentanone	Leaves	[41]
68	2-Nonyne	Leaves	[41]
69	3,5-Diethyl-toluene	Leaves	[40]
70	3-Octanol	Leaves	[45]
71	4,4-Dimethyl-2-cyclopenten-1-one	Leaves	[41]
72	4-Tert-pentylphenol	Leaves	[40]
73	Acetophenone	Leaves	[40]
74	Acetyl eugenol	Leaves	[40]
75	a-Cubebene	Leaves	[40]
76	Alloaromadendrene	Leaves	[41]
77	All-*trans*-squalene	Leaves	[40]
78	Anisole	Stems	[7]
79	Apiol	Leaves	[40,41]
80	Asarone	Leaves	[7,41]
81	*a*-Terpinyl acetate	Leaves	[43]
82	Benzaldehyde	Leaves	[40,41,44]
83	Benzene acetaldehyde	Leaves	[40]
84	Bornyl acetate	Leaves	[40,45]
85	Cadina-3,9-diene	Leaves	[40]
86	Calarene	Leaves	[41]
87	Camphane	Leaves	[41]
88	Camphene	Leaves	[40,41,43]
89	Carvone	Leaves	[45]
90	Caryophyllene	Stems, Leaves	[7,40,41,45]
91	Caryophyllene oxide	Leaves, Seeds	[7,40,41,43,44,45]
92	*cis*-Pyranoid	Leaves	[45]
93	*cis*-(Z)-α-Bisabolene epoxide	Leaves	[41]
94	*cis*-Asarone	Leaves	[41]
95	*cis*-Geranio	Leaves	[40]
96	*cis*-Lanceol	Leaves	[40]
97	*cis*-Nerolidol	Leaves	[41]
98	*cis*-Ocimene	Leaves	[41]
99	*cis*-Verbenol	Leaves	[41]
100	Cosmene	Leaves	[41]
101	Cuminaldehyde	Leaves	[43]
102	Curlone	Stems	[7]
103	Cycloheptane	Leaves	[41]
104	Cyclohexanone	Leaves	[41]
105	Decane	Leaves	[41]
106	Dihydrocarveol	Leaves	[41,45]
107	Dihydrocarveol acetate	Leaves	[41,45]
108	Dodecane	Leaves	[41]
109	Egomaketone	Leaves	[45,46]
110	Elemicin	Leaves	[29,41,45]
111	Elixene	Leaves	[7]
112	Elsholtziaketone	Leaves	[44]
113	ɛ-Muurolene	Leaves	[41]
114	Eremophilene	Leaves	[41]
115	Eucalyptol	Leaves	[41,45]
116	Farnesol	Leaves	[41,43]
117	Furfuryl alcohol	Leaves	[41]
118	Geraniol	Leaves	[41]
119	Germacrene D	Leaves	[40,41,44]
120	Germacrene D-4-ol	Leaves	[40]
121	Heneicosane	Leaves	[41]
122	Hexadecane	Leaves	[41]
123	Hexahydro farnesyl acetone	Leaves	[44]
124	Humulene epoxide II	Leaves	[41,45]
125	Humulene epoxide-II	Leaves	[44]
126	Isobornyl acetate	Leaves	[40]
127	Isocaryophyllene	Leaves	[41]
128	Isoegomaketone	Leaves	[44]
129	Isoelemicin	Leaves	[41]
130	Isoeugenol	Leaves	[41]
131	Isolimonene	Leaves	[41]
132	Isomenthone	Leaves	[41]
133	Isopulegone	Leaves	[41]
134	Laurolene	Leaves	[41]
135	Limonen oxide, *cis*	Leaves	[45]
136	Limonene	Leaves	[29,40,41,43,45]
137	Limonene oxide	Leaves	[45]
138	Limonene oxide, *trans*	Leaves	[45]
139	Linalool oxide	Leaves	[45]
140	Linalool oxide *trans*	Leaves	[45]
141	Linalyl oxide *cis*	Leaves	[45]
142	Longifolene	Leaves	[41]
143	Longipinocarvone	Leaves	[40]
144	Massoia lactone	Leaves	[41]
145	Menthol	Leaves	[41,47]
146	Menthone	Leaves	[41]
147	Methyl chavicol	Leaves	[43]
148	Methyl eugenol	Leaves	[41]
149	Methyl geranate	Leaves	[40,41,45]
150	Methyl heptenone	Leaves	[41]
151	Methyl isoeugenol	Leaves	[41]
152	Methyl thymyl ether	Seeds	[7]
153	M-Mentha-6,8-diene	Leaves	[40]
154	Myrcene	Leaves	[41,45]
155	Myristicin	Leaves	[29,40,41,45]
156	Naginata ketone	Leaves	[41]
157	Nerol acetate	Leaves	[41]
158	*n*-Heptadecane	Leaves	[41]
159	Nonacosane	Leaves	[40]
160	Nonane	Leaves	[41]
161	*n*-Tricosane	Leaves	[41]
162	Octacosane	Leaves	[40]
163	Patchoulane	Leaves	[40,41]
164	*p*-Cymene	Seeds	[7]
165	Pentacosane	Leaves	[41]
166	Perilla ketone	Leaves	[29,40,44,45,46,47,48]
167	Perillaldehyde	Leaves	[40,41,43,45]
168	Perillene	Leaves	[41,44]
169	Perillic acid	Leaves	[49]
170	Perillyl alcohol	Leaves	[40,45]
171	Piperitenone	Leaves	[41]
172	*p*-Menth-1-en-4-ol	Leaves	[40]
173	*p*-Menth-1-en-8-ol	Leaves	[40]
174	*p*-mentha-2,8-dione	Leaves	[43]
175	*p*-Mentha-3,8-diene	Leaves	[41]
176	Pseudolimonene	Leaves	[41]
177	Pulegone	Leaves	[41]
178	Phthalic acid	Stems	[7]
179	Phytol	Leaves	[7]
180	Phytone	Leaves	[41]
181	Sabinene	Leaves	[40,41,43]
182	Santolina triene	Leaves	[41]
183	Spathulenol	Leaves	[7,40,44]
184	Styrene	Leaves	[41]
185	Terpinen-4-ol	Leaves	[41]
186	Terpinolene	Leaves	[40,41,43,45]
187	Thujyl alcohol	Leaves	[41]
188	*trans*-Furanoid	Leaves	[45]
189	*trans*-Nerolidol	Leaves	[7,40,41,46]
190	*trans*-Shisool	Leaves	[40]
191	Triacontane	Leaves	[40]
192	Tridecane	Leaves	[41]
193	Valencene	Leaves	[41]
194	Valeric acid, pent-2-en-4-ynyl ester	Leaves	[40]
195	Viridiflorene	Leaves	[41]
196	Viridiflorol	Leaves	[41]
197	α-Bergamotene	Seeds	[7]
198	α-Bulnesene	Leaves	[41]
199	α-Cadinol	Leaves	[40,41]
200	α-Caryophyllene	Leaves	[40,43]
201	α-Caryophyllene	Leaves	[41]
202	α-Citral	Leaves	[40,41]
203	α-Copaene	Leaves, Seeds	[7,40,41,44]
204	α-Curcumene	Stems	[7]
205	α-Farnesene	Leaves, Seeds	[7,29,41,43]
206	α-Fenchene	Leaves	[41]
207	α-Patchoulene	Leaves	[41]
208	α-Pinene	Leaves	[40,41,43]
209	α-Santalol	Leaves	[41]
210	α-Terpineol	Stems	[7]
211	α-Terpineol	Leaves	[41]
212	β-Cubebene	Leaves	[43]
213	β-Bourbonene	Leaves	[40,41]
214	β-Cadinene	Leaves	[41]
215	β-Caryophyllene	Stems, Leaves, Seeds	[7,29,41,43,44,45,46]
216	β-Citronellene	Leaves	[41]
217	β-Cyclocitral	Leaves	[45]
218	β-Dehydroelsholtziaketone	Leaves	[41]
219	β-Elemene	Leaves	[7,40,41]
220	β-Farnesene	Stems, Leaves, Seeds	[7,29,41]
221	β-Guaiene	Leaves	[40]
222	β-Gurjunene	Leaves	[41]
223	β-Ionone	Leaves	[41]
224	β-Linalool	Leaves	[7,29,40,41,44,46]
225	β-Murolene	Leaves	[45]
226	β-Pinene	Leaves	[40,41,43]
227	β-Phellandrene	Leaves	[40,41]
228	β-Selinene	Leaves	[41]
229	β-Terpinene	Leaves	[41]
230	γ-Pyronene	Leaves	[41]
231	δ-Cadinene	Leaves	[41]
232	δ-Elemene	Leaves	[40,41]

**Table 3:** Triterpenes, phytosterols, fatty acids, polycosanol, and tocopherol compounds found in *Perilla frutescens* species.

Number	Compounds	Parts	References
**Triterpenes**			
233	3-Epicorosolic acid	Leaves	[54]
234	3-Epimaslinic acid	Leaves	[54]
235	Augustic acid	Leaves	[54]
236	Corosolic acid	Leaves	[54]
237	Hyptadienic acid	Leaves	[54]
238	Oleanolic acid	Leaves	[50,54]
239	Pomolic acid	Leaves	[54]
240	Tormentic acid	Leaves	[50,54]
241	Ursolic acid	Leaves	[50,54]
**Phytosterols**			
242	Campesterol	Seeds	[51]
243	Oxalic acid	Leaves	[55]
244	Stigmasterol	Seeds	[51]
245	Triacylglycerols	Seeds	[56]
246	β-Amyrin	Seeds	[51]
247	β-Sitosterol	Seeds	[51]
**Fatty acids**			
248	5α-Cholestane	Seeds	[51,53]
249	Arachidic acid	Seeds	[51]
250	Eicosenoic acid	Seeds	[51]
251	Lauric acid	Seeds	[47]
252	Linoleic acid	Seeds	[3,47,51,53,57,58]
253	Linolenic acid	Seeds, Leaves	[3,47,51,53,57,58]
254	Oleic acid	Seeds	[3,40,47,51,57,58]
255	Palmitic acid	Seeds, Leaves	[3,40,47,51,53,57,58]
256	Pentadecanoic acid	Seeds	[51]
257	Stearic acid	Seeds	[3,47,51,53,57,58]
258	β-Cholestanol	Seeds	[51]
**Polycosanol**			
259	Docosanol	Seeds	[51]
260	Eicosanol	Seeds	[51,56,59]
261	Heneicosanol	Seeds	[51]
262	Heptacosanol	Seeds	[51]
263	Hexacosanol	Seeds	[51]
264	Octacosanol	Seeds	[51]
265	Tetracosanol	Seeds	[51]
266	Triacontanol	Seeds	[51]
267	Tricosanol	Seeds	[51]
**Tocopherols**			
268	α-Tocopherol	Seeds	[51]
269	β-Tocopherol	Seeds	[51]
270	γ-Tocopherol	Seeds	[51]
271	δ-Tocopherol	Seeds	[51]

## Abbreviations

PF	Perilla frutescens
PFL	Perilla frutescens leaf
pg/mL	Picogram/millilitre
UV	Ultraviolet–visible spectroscopy
NMR	Nuclear magnetic resonance spectroscopy
ESI-MS	Electrospray ionisation mass spectrometry
IT-TOF MS	Ion-trap time-of-flight mass spectrometry
Eos	Essential oils
SFE-CO2	Supercritical fluid extraction
HS-SPME	Headspace solid phase microextraction
HPLC	High-performance liquid chromatography
TLC	Thin layer chromatography
GC-MS	Gas chromatography–mass spectrometry
ROS	Reactive oxygen species
DPPH	2,2-Diphenyl-1-picryl-hydrazyl-hydrate
ABTS	2,2′-Azino-bis(3-ethylbenzothiazoline-6-sulphonic acid)
IC_50_	Half-maximal inhibitory concentration
RA	Rosmarinic acid
LPS	Lipopolysaccharide
d-GalN	D-galactosamine
TNF-alpha	Tumor necrosis factor-alpha
LDL	Low-density lipoprotein
DDC	2′,3′-Dihydroxy-4′,6′-dimethoxychalcone
ARE	Antioxidant response element
MIC	Minimum inhibitory concentration
CFU/mL	Colony-forming units per millilitre
TSST-1	Toxic shock syndrome toxin 1
anti-DNP IgE	Dinitrophenyl Immunoglobulin
DNFB	2,4-Dinitrofluorobenzene
C57BL/6	(C57 black 6) is a common inbred strain of laboratory mouse.
MMP-9	Matrix metallopeptidase 9
IL-31	Interleukin 31
TH2 T	Helper 2
PCA	Passive cutaneous anaphylaxis
PMNL	Polymorphonuclear leukocytes
FST	Forced swim test
CUMS	Chronic unexpected mild stress
BDNF	Brain-derived neurotrophic factor
Inos	Inducible nitric oxide synthase
MAPK	Mitogen-activated protein kinase
NF-κB	Nuclear factor-kappa
ALA	α-Linolenic acid
HMGB1	High mobility group box 1 protein
CLP	Cecal ligation and puncture
TPA	Tetradecanoylphorbol-13-acetate
EBV-EA	Epstein–Barr virus early antigen
IBD	Inflammatory bowel disease
STAT3	Signal transducer and activator of transcription 3
DMBA	7,12-Dimethylbenz[a]anthracene
Hep G2	Liver hepatocellular cells (a human liver cancer cell line)
IK	Isoegomaketone
ALT	Alanine transaminase
AST	Aspartate aminotransferase
SOD	Sphincter of oddi dysfunction
